# Prospects of anti-GD2 immunotherapy for retinoblastoma

**DOI:** 10.3389/fimmu.2024.1499700

**Published:** 2024-11-15

**Authors:** Xinlong Zhang, Wulin You, Yuntao Wang, Rebeka Dejenie, Chenhao Wang, Yan Huang, Jingjing Li

**Affiliations:** ^1^ Affiliated Hospital of Shandong Second Medical University,School of Clinical Medicine, Shandong Second Medical University, Weifang, Shandong, China; ^2^ Jinming Yu Academician Workstation of Oncology, Shandong Second Medical University, Shandong, China; ^3^ School of Clinical Medicine, Shandong Second Medical University, Weifang, China; ^4^ Department of Orthopedics, Wuxi Hospital Affiliated of Nanjing University of Chinese Medicine, Wuxi, China; ^5^ Medical Center, University of Chicago, Chicago, IL, United States; ^6^ School of Medicine, University of California, Davis, Davis, CA, United States

**Keywords:** retinoblastoma, GD2, anti-GD2 monoclonal antibody, GD2-CAR cell therapy, immunotherapy

## Abstract

Retinoblastoma is the most common type of eye tumor in infants and children. Current treatments for retinoblastoma include intravenous chemotherapy, intra-arterial chemotherapy, intravitreal chemotherapy, cryotherapy, radiotherapy, and surgery. However, these treatments come accompanied by adverse effects such as the toxic side effects of chemotherapeutic drugs, post-operative complications including blindness after surgery, or other complications caused by radiotherapy. Immunotherapy is more promising for its low toxicity on normal cells and effectively improves the quality of life of patients. Disialoganglioside (GD2), a sphingolipid expressed on the surface of retinoblastoma, is a potential therapeutic target for retinoblastoma. We summarized immunotherapeutic approaches for both preclinical studies and clinical trials of GD2. An anti-GD2 monoclonal antibody (Dinutuximab), which has been approved for the treatment of high-risk neuroblastomas, has shown promising efficacy in improving patients’ prognosis. Additionally, chimeric antigen receptors (CAR)-T therapy, GD2 vaccines and nanoparticles are also potential therapeutics. Finally, we discuss the prospects and current limitations of these immunotherapeutic approaches for treating retinoblastoma, as well as how to address these problems.

## 1 Introduction

Retinoblastoma (RB) is the most common type of intraocular malignancy in infancy and childhood, usually caused by a mutation in the retinoblastoma 1 (*RB1*) gene on chromosome 13, which occurs in one or both eyes (1). The survival rate of children with retinoblastoma varies in countries with different income levels, depending on early diagnosis, staging, and treatment options. In high-income countries, the 3-year survival rate can reach more than 99%. In contrast, in low-income countries, many children present to the clinic with disseminated lesions and some with distant metastases, resulting in a 3-year survival rate of less than 58% (2, 3). It is not satisfactory that there are essentially no treatment options available for children with disseminated retinoblastoma (4). Therefore, new therapeutic approaches are desperately needed for both intraocular and extraocular illnesses.

The blood-retinal barrier (BRB), which prevents the interchange of macromolecules between the retina and the circulatory system, is considered to keep retinoblastoma separate from blood cells, in contrast to the majority of malignancies that frequently interact with the vascular system (5, 6). Immunohistochemistry of retinoblastoma and immunocytochemical analysis of tumor-infiltrating vitreous samples detected immune cells but not in normal retinal tissue or normal vitreous samples, suggesting that destruction of the BRB by retinoblastoma cells promotes immune cell infiltration and disrupts immune homeostasis (7).

Current treatments for retinoblastoma include intravenous chemotherapy, intra-arterial chemotherapy, intravitreal chemotherapy, cryotherapy, radiotherapy, and surgery (8, 9). Intravenous chemotherapy not only controls the tumor, but also plays a role in preventing distant metastases (10). However, it is difficult to avoid complications such as alopecia and ototoxicity with intravenous chemotherapy (8, 11). In contrast, intra-arterial chemotherapy can lead to a higher concentration of locally delivered chemotherapeutic drugs and better control of ocular tumors (12). Although intra-arterial chemotherapy also results in reduced systemic complications, it causes complications localized to the eye including periorbital edema, periocular congestion, and vitreous hemorrhage (13). Intravitreal chemotherapy also directly delivers chemotherapeutic drugs to the tumor lesion, which plays a very good therapeutic effect on patients with intravitreal dissemination (8). Cryotherapy as a local treatment is usually combined with intravenous chemotherapy or intra-arterial chemotherapy. However, this local treatment can cause choroidal retinal scarring and even lead to vision loss (8). Radiation therapy is now generally considered as a treatment of last resort, which is related to its complication rate, as external radiation radiotherapy (EBRT) can cause cataracts, orbital hypoplasia, and in severe cases, secondary tumors (14). Some advanced patients must undergo surgical removal to prevent metastasis of the tumor, but postoperatively, patients may face physical effects such as postoperative hemorrhage, infection, and psychological effects such as anxiety and depression (15). With the advent of systemic chemotherapy, the use of external beam radiation therapy has been gradually relinquished. Although novel methods for delivering chemotherapeutic agents to tumor sites are now available, the toxicity associated with chemotherapy remains a significant concern. Thus, there is an urgent need to develop new targeted therapies that can preserve patients’ vision and improve their overall quality of life (16).

In recent years, targets related to RB immunotherapy include GD2 (17), PD-1 (18), B7H3 (19), EpCAM (20), SYK (21). With high density in tumor cells and limited expression in normal tissues, GD2 is ideally suited as a target for cancer therapy, and has been ranked by the US National Cancer Institute as one of the most promising anticancer therapies accordingly (22). Malignant transformations are commonly accompanied by alterations in cell surface glycosylation. GD2 has been identified as a potential serum marker for retinoblastoma since as early as 1993 (23). GD2 has served as an effective target on human retinoblastoma cell lines (17). This review focuses on ganglioside GD2 as a therapeutic target for retinoblastoma and the therapeutic approaches being investigated.

## 2 Disialoganglioside (GD2) in retinoblastoma

### 2.1 Introduction of GD2

**Figure 1:**
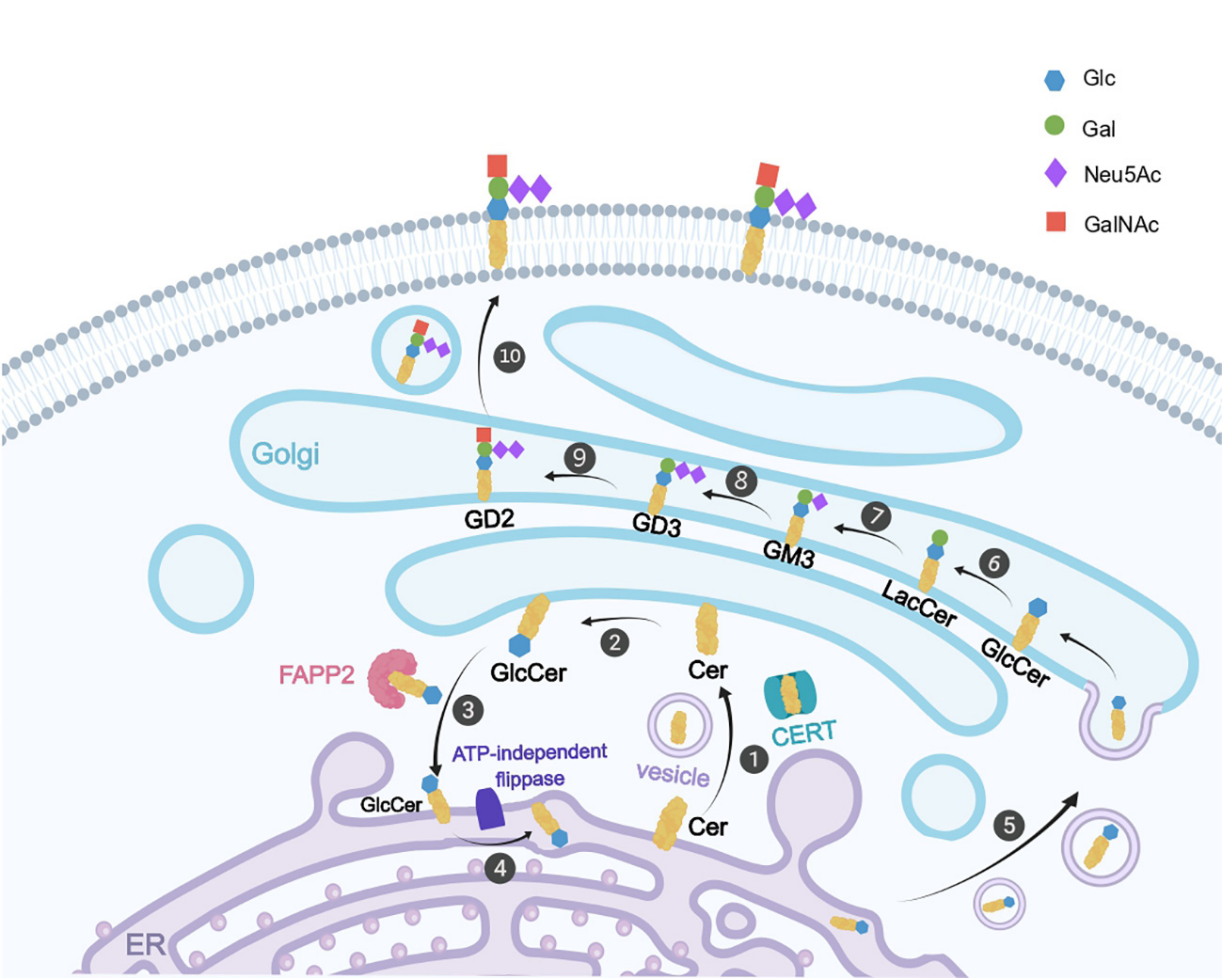
The biosynthetic process of GD2. Ceramide (Cer) is first synthesised in the endoplasmic reticulum (ER), ① Cer transfers to the inner Golgi (cytoplasmic side) via vesicles or ceramide transfer protein (CERT), ② Cer binds to glucose to produce glucosylceramide (GlcCer), ③ GlcCer is transferred to the cytoplasmic face of the ER via FAPP2 (phosphatidylinositol 4-phosphate adaptor protein), ④ GlcCer is flipped to the luminal side of the ER by ATP-independent flippase, ⑤ GlcCer in the lumen of the ER is translocated to the Golgi via vesicles, ⑥ GlcCer binds to galactose in the Golgi to form lactosylceramide (LacCer), ⑦ LacCer binds to salivary acid (Neu5Ac) to produce GM3, ⑧ GM3 continues to bind to Neu5Ac to generate GD3, ⑨ GD3 binds to N-acetylgalactosamine (GalNAc) to produce GD2, ⑩ GD2 is transferred to the cell membrane via vesicles secreted by the Golgi apparatus.

GD2 is enmeshed with its ceramide tail (fatty acid-linked sphingosine) in the cell membrane (24, 25). The sugar moiety, which is made up of galactose (Gal), N-acetylgalactosamine (GalNAc), and glucose (Glc; connected to ceramide), is exposed to the extracellular milieu and has antigenic properties that promote mutual recognition and adhesion between cells (25, 26). Gal is formed by two more sialic acid residues (N-acetylneuraminic acid, NeuAc), which also give GD2 a negative charge (25). Gangliosides first synthesize ceramide in the endoplasmic reticulum (27), which is subsequently transferred to the Golgi apparatus via ceramide transfer protein or vesicles (28, 29) and glycosylated under the action of glycosyltransferase and sialyltransferase (30). The glycosylated and sialylated ceramides are then transferred to the plasma membrane via the cytosolic action of the Golgi apparatus by the transfer of vesicles (**Figure 1**) (31). Ceramide binds to glucose in the presence of UDP-glucose-ceramide-glucosyltransferase, resulting in glucosylceramide (GlcCer). The GlcCer combines with galactose in the presence of lactosylceramide synthetase to produce lactosylceramide (LacCer), which then combines with salivary acid to form GM3. In the presence of GD3 synthetase, GM3 continues to bind to salivary acid to produce GD3, which then binds to N-acetylgalactosamine (GalNAc) to produce GD2 (**Figure 1**) (32). Sialic acids can be further O-acetylated (33), allowing the O-acetylated derivative of GD2, O-acetyl-GD2, to gain attention as a novel antigen targeting GD2-positive cancers (34). O-acetyl-GD2 is coexpressed with GD2 in a variety of tumor cells including lung carcinoma, melanoma, osteosarcoma, brain tumors, and neuroblastoma (35, 36).

The catabolism of GD2 begins with the formation of endosomes by endocytosis, which then fuses with lysosomes to form invaginated vesicles. The outer layer of these vesicles carries GD2, which is then degraded when exposed to lysosomal matrix glycohydrolases (37). GD2 removes one salivary acid in the presence of sialidase to form GM2, then removes N-acetylgalactosamine in the presence of hexosaminidase to form GM3 (24). The remaining salivary acid is removed in the presence of SAP-B and sialidase to form LacCer, which is subsequently degraded to ceramide in the sequential presence of β-galactosidase, β-glucosidase, and SAP-C. This is ultimately broken down into sphingomyelin base and fatty acid in the presence of ceramidase and SAP-D (24, 37).

Several glycosyltransferase (GT) genes implicated in GD2 biosynthesis (*ST3GAL5*, *B4GALNT1*, and *ST8SIA1*) were found to be substantially elevated in cancer stem cells (CSCs) by gene expression analysis (38, 39). GD2 expression was decreased by *ST8SIA1* knockdown, which changed the phenotype from CSCs to non-CSCs, thereby inhibiting tumor formation *in vivo*.

Before birth, GD2 is expressed in neural and mesenchymal stem cells (40). Whereas after birth, it is expressed in the central nervous system (41), peripheral nerves (41), dermal melanocytes (41), and mesenchymal stromal cells (42, 43). However, the normal human retina does not exhibit GD2 expression (44, 45). The high expression level of GD2 is associated with reduced apoptosis and enhanced proliferation, adhesion, angiogenesis, migration and invasion of tumor cells in a variety of solid tumors, including retinoblastoma (RB) (23, 45–48), neuroblastoma (NB) (41, 49), breast cancer (BC) (38, 50), bladder cancer (BLCA) (51), small cell lung carcinoma (SCLC) (52, 53), osteosarcoma (OS) (54), ewing sarcoma (ES) (47, 55, 56) and diffuse intrinsic pontine glioma (DIPG) (57). The tumor stage and proliferation of retinoblastoma are both positively correlated with GD2 expression (7). GD2 is also detectable in the serum (23), bone marrow (48), and cerebrospinal fluid (58) of patients with extraocular dissemination.

### 2.2 Functions of GD2

GD2 activates receptor tyrosine kinase (RTK)-mediated signaling and engages in cell proliferation and migration, angiogenesis, and tumor metastasis (59). GD2 is directly involved in RTK activation, possibly realized by specific interaction of the oligosaccharide portion of GD2 with the RTK receptor (60). The activation of c-Met was found to be associated with GD2 (60, 61), which in turn activated the MEK/Erk and PI3K/Akt signaling pathways, resulting in enhanced cell migration, proliferation, and tumor growth (60). Moreover, carbohydrate interactions between GD2 and c-Met induce constitutive activation of c-Met even in the absence of hepatocyte growth factor (62). Additionally, GD2 is physically associated with integrins on the same cell (61, 63), which are strongly associated with focal adhesion kinase (FAK) both physically and functionally. The formation of a tertiary complex consisting of GD2, integrins, and FAK further generates, and/or maintains the malignant properties of SCLC cells by activating MAPK signaling (63). In addition, associations between GD2 and the FAK/AKT signaling pathway were found in melanoma cells, glioma cells, prostate cancer cells, triple-negative breast cancer cells, and osteosarcoma cells with high GD2 expression (24).

The expression of GD2 is increased when epithelial-mesenchymal transition (EMT) is induced (38). Studies have shown that in bladder cancer, the EMT process could be reversed upon inhibition of GD2 synthesis (51). Additionally, GD2 might have indirect impacts on the development of tumors. It has been noted to suppress human dendritic cells (64) and T-cell immune responses (24, 65), most likely via myeloid-derived suppressor cells (66) and regulatory T cells (67). Moreover, GD2-positive melanoma cells produce small extracellular vesicles (sEVs), which enable nearby GD2-negative melanoma cells to take on more malignant characteristics, such as cell proliferation, invasion and adhesion (68).

GD2 has been used as a biomarker of retinoblastoma (23, 48, 58, 69), aggressive high-grade bladder cancer (51), and a CSC-specific cell surface marker in breast cancer (38). Additionally, correlation has been verified between GD2 expression and lung cancer malignant phenotypes (70). Moreover, GD2 synthase serves as a marker for minimal disseminated disease in retinoblastoma patients (69, 71–73).

## 3 GD2 targeting monoclonal antibodies

**Figure 2:**
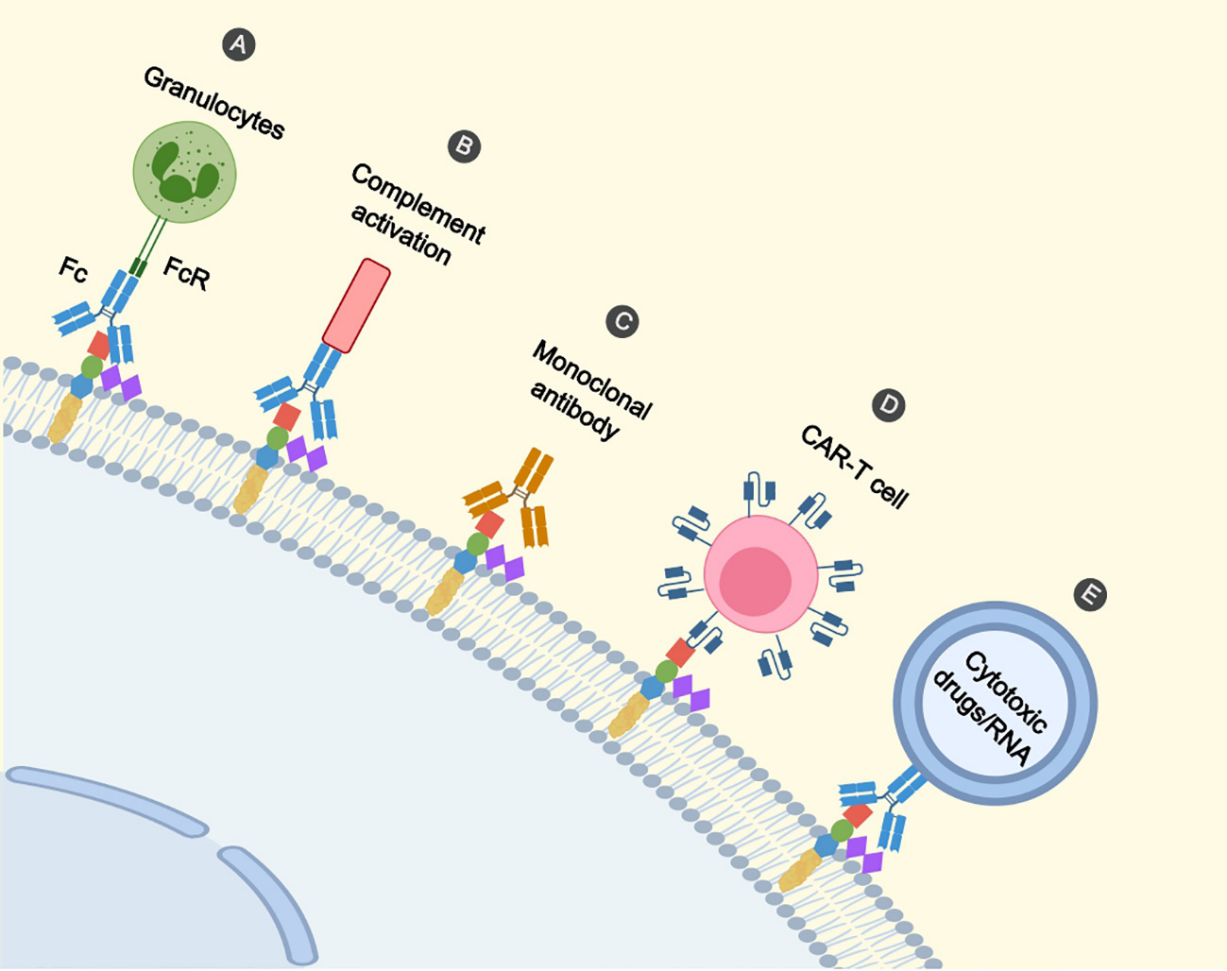
Immunotherapeutic mechanisms on GD2. **(A)** Binding of the Fc segment of the antibody to the FcR of the effector cell (e.g. granulocyte) initiates the killing of the cell. **(B)** Antibodies mediate tumour cell lysis via complement-dependent cytotoxicity. **(C)** Antibodies directly induce cell death. **(D)** CAR-T cells recognize GD2 and promote cell lysis. **(E)** Nanoparticles recognize GD2 by antibodies and deliver cytotoxic drugs and RNA to tumour cells.

The blood-brain barrier (BBB) prevents intravenous anti-GD2 mAbs from entering the central nervous system (CNS), making GD2 an ideal target for CNS extranodal neuroectodermal tumors (74). Three mechanisms of action have been identified for anti-GD2 monoclonal antibody (mAb) against tumor cells expressing GD2 (26):

Firstly, anti-GD2 mAbs initiate phagocytosis of tumor cells by macrophages, destruction of tumor cells by natural killer (NK) cells, and granulocyte-mediated cytotoxicity (**Figure 2A**) (75, 76). Siglec (sialic acid-binding immunoglobulin-like lectin)-7 is an inhibitory receptor expressed on immune cells, inhibits immune cell activity through the cytoplasmic immunoreceptor tyrosine-based inhibitory motif (ITIM) domain (77). Siglec-7 is a ligand for GD2 and binds to GD2 to inhibit immune cell activity via ITIM. Anti-GD2 antibody blocks GD2 from binding to Siglec-7 so that immune cells are not suppressed.

Furthermore, anti-GD2 mAbs mediate the lysis of tumor cells through complement-dependent cytotoxicity (**Figure 2B**) (76, 78). Besides, it was shown that cell death directly induced by anti-GD2 mAb was associated with a mitochondria-dependent pathway. Anti-GD2 mAb interacted with GD2 to cause rapid hyperpolarization of the membrane potential of mitochondria, followed by apoptotic volume decrease (AVD) and alteration of cell membrane permeability, suggesting that anti-GD2 mAb-induced cell death is characterized by apoptosis and necrosis (**Figure 2C**) (79). We summarize some of the studies on anti-GD2 monoclonal antibodies (**Table 1**).

**Table 1:** lists studies on the anti-GD2 monoclonal antibody collected from https://clinicaltrials.gov/.

Agent	Clinical Trial Number	tumour	phase	status	Description of the study
hu14.18K322A	NCT01857934	Neuroblastoma	II	Active, not recruiting	This study will be a pilot Phase II study of a unique anti-disialoganglioside (anti-GD2) monoclonal antibody (mAb) called hu14.18K322A given with induction chemotherapy.
m3F8	NCT00492167	Neuroblastoma	I	Completed	This phase I trial investigated the side effects and optimal dose of B-glucan in combination with the monoclonal antibody 3F8 in patients with metastatic neuroblastoma.
Ch14.18	NCT00005576	Neuroblastoma	I	Completed	This phase I trial investigates the maximum tolerated dose of Ch14.18 in combination with sargramostim and interleukin-2 in children with neuroblastoma who have completed bone marrow or peripheral blood stem cell transplantation.
Ch14.18/CHO	NCT01704872	Neuroblastoma	I	Completed	The primary objective of this Phase I clinical trial was to reassess the toxicity of ch14.18/CHO, and the secondary objectives were to determine pharmacokinetics and immunostimulation
dinutuximab	NCT05400603	Neuroblastoma	I	Recruiting	This clinical phase I trial treats children with refractory, recurrent or progressive neuroblastoma with yo T cells in combination with Dinutuximab, Temozolomide, Irinotecan and Zoledronate.
naxitamab	NCT05489887	Neuroblastoma	II	Recruiting	The Phase II clinical trial evaluated the efficacy and safety of the antibody with an induction regimen by adding Naxitamab to induction chemotherapy in patients with newly diagnosed high-risk neuroblastoma.
dinutuximab beta	NCT05080790	Leiomyosarcoma	II	Recruiting	The study focuses on evaluating the feasibility of combination therapy by treating patients with metastatic or inoperable smooth muscle sarcoma with Dinutuximab Beta, Zoledronic Acid and Low-dose Interleukin (IL-2).

### 3.1 Main types of anti-GD2 mAbs approved for treatment

Dinutuximab (Ch14.18/SP2/0): Dinutuximab binds specifically to GD2 and is the first GD2 monoclonal antibody licensed for the treatment of high-risk neuroblastoma (HRNB) (80). The Children’s Oncology Group (COG) conducted a large randomized trial in 2010, which revealed that immunotherapy with the addition of ch14.18, alternating with granulocyte–macrophage colony-stimulating factor(GM-CSF) and interleukin-2, significantly improved outcome as compared with standard therapy in patients with HRNB (81) with similar long-term follow-up results (82).

Dinutuximab beta: Dinutuximab beta is an analog of Dinutuximab, which has been approved for use as frontline post-consolidation therapy (81) and as a second-line drug to improve survival chances in patients with HRNB (83, 84).

Naxitamab (humanized anti-GD2 mAb, Hu3F8): Naxitamab is an FDA-approved anti-GD2 monoclonal antibody that is being used in combination with GM-CSF in children and adults with HRNB (85), and is associated with modest toxic effects, low immunogenicity, and substantial anti-neuroblastoma activity (86).

### 3.2 Human/mouse chimeric anti-GD2 mAbs

Dinutuximab binding to CD16-expressing NK-92MI cells incites strong antitumor effects on RB cell. Mechanism analysis shows antibody-dependent cell-mediated cytotoxicity (ADCC) is increased, meanwhile, the perforin-granzyme cytolytic pathway is activated by NK cells (7).

A combination of Dinutuximab and anti-CD47 antibodies results in the recruitment of tumor-associated macrophages (TAMs) to mediate robust and durable anti-tumor responses, which might expand the clinical use of anti-GD2 mAbs to other GD2^+^ diseases beyond NB (77). Potent synergy for the combination of Dinutuximab and anti-CD47 has been established in a xenograft mouse model of NB, where the combination eradicates tumors (77). Additionally, a similar effect was seen in small cell lung cancer, where the combination significantly reduced tumor burden and extended survival (77).

Recently, a novel IgA anti-GD2 immunotherapy (IgA3.0 ch14.18) was developed, which could replace Dinutuximab to treat HRNB since it provides similar efficiency but does not induce neuropathic pain (87). Moreover, it offers an extended half-life, and enhanced stability, and may reduce the risk of developing potential side effects, such as Berger’s disease (87).

### 3.3 Humanized anti-GD2 mAbs

Compared to Dinutuximab, a humanized anti-GD2 monoclonal antibody with the K322A mutation (hu14.18K322A), is effective in treating refractory or recurrent neuroblastoma, potentially reducing pain and other side effects (88). Currently, hu14.18K322A has entered phase II clinical trials and has been shown to significantly improve early response and outcomes in children with newly diagnosed HRNB (89).

To enhance anti-tumor efficacy, an immunocytokine (IC) was developed by linking interleukin (IL)-2 to the COOH terminus of a humanized anti-GD2 mAb (hu14.18-IL2) (90, 91). Recently, Nguyen, R. et al. developed two new ICs, hu14.18-IL15 and -IL21 and demonstrated their superior antibody-dependent cell-mediated cytotoxicity compared to hu14.18-IL2 in fully immunocompetent syngeneic mouse models with orthotopic NB. This ultimately led to complete tumor regression and long-term survival beyond 100 days (92). IL15 and IL21 act on both CD8+ T cells and NK cells (93). Moreover, IL15 also acts on the myeloid compartment by decreasing the recruitment of polymorphonuclear/granulocytic myeloid-derived suppressor cells to the tumor microenvironment and increasing pro-inflammatory M1-like TAMs (92).

Radiopharmaceutical therapy (RPT) is a type of radionuclide labeling associated with monoclonal antibodies. It has been shown that actinium-225 radiolabels humanized anti-GD2 mAb (hu3F8), which is specifically targeted to the tumor site by an antibody, causes an ionization event that damages the double-stranded DNA of the tumor cells (94). Hu3F8-based alpha-particle therapy has been proven to be a safe and effective approach for OS. Liatsou, I et al. demonstrated the feasibility of such therapy by treating both an orthotopic canine OS xenograft mouse model and two dogs with spontaneously occurring OS, using an alpha-particle emitter labeled Hu3F8 (94). Moreover, hu3F8 labeled with Indium-111 enables good imaging of human and canine OS patients (95).

### 3.4 Major challenges for anti-GD2 mAbs

Treatment with anti-GD2 antibodies is associated with a temporary painful neuropathy syndrome in patients (81), which could be attributed to the antibodies’ ability to bind to the GD2^+^ myelin sheath of nerve fibers (96, 97), complement fixation (97) and direct electrophysiologic effects on nerve fibers (98).

At least 40% of NB patients relapse despite having received anti-GD2 mAbs during upfront therapy (81, 99). Killer immunoglobulin-like receptors (KIR) could affect NK cell activity which in turn affects patients’ response to immunotherapy. Recurrent HRNB Patients with a KIR2DL2+/ligand+ genotype or a KIR3DL1+/ligand+ genotype had better survival outcome in the setting of GD2-directed immunotherapy (100), shedding light on therapy selection in the setting of relapsed and refractory disease.

More than 40% of NB patients fail to respond or develop resistance to anti-GD2 mAbs (101). Recently, two mechanisms for the development of anti-GD2 mAbs resistance have been identified. Firstly, GD2 expression is reduced when NB patients relapse (102). Adeiye A et al. found that NB patients with high expression of YAP (yes-associated protein) had high resistance to GD2 immunotherapy (103). Mechanistically, YAP transcriptionally suppresses *ST8SIA1* that encodes GD3 synthase, the rate-limiting enzyme for GD2 synthesis, suggesting that YAP could serve as both a mediator and potential biomarker of anti-GD2 mAb resistance. Additionally, NB-derived small extracellular vesicles (sEVs) induce resistance to Dinutuximab by promoting an immunosuppressive tumor microenviroment characterized by a decrease in tumor-infiltrating NK cells and an increase in TAMs (104). Meanwhile, Tipifarnib, a farnesyltransferase inhibitor, enhances the efficacy of Dinutuximab by inhibiting sEV secretion (104). Given that Tipifarnib has entered phase II clinical trials in pediatric patients with advanced solid tumors, the combination of Dinutuximab and Tipifairnib could be rapidly translated to the clinic for HRNB patients.

## 4 GD2-CAR cell therapy

Chimeric antigen receptors(CAR)-T therapy has shown great promise in relapsed or refractory B-cell cancers (105, 106). However, in solid tumors, only a minority of patients have a good prognosis (107–109). This is mainly due to the limited persistence of CAR-T cells *in vivo* and impaired T-cell function when treating solid tumors (110). In four trials, a cumulative total of 42 patients with active NB were treated with GD2-CAR T-cell-based therapy, of which only three achieved a sustained objective response (111–114).

Similarly, NK cells have been applied in various types of CARs immunotherapy against both hematological malignancies (115) and solid tumors (116–121). Preclinical and clinical trials using CAR NK cells have demonstrated that CAR NK cells have several advantages over CAR T cells, such as increased general availability of modified immune cells from healthy donors, potent anti-tumor effects, and therapeutic safety (122–126).

### 4.1 Recent advances on GD2-CARs in RB

GD2 has been suggested as a possible target for RB-specific CAR-T cell treatment by Andersch, L et al. (**Figure 2D**) (17). RB cells are effectively targeted and killed *in vitro* by GD2-CAR T cells (17, 44) in a manner dependent on antigen density and cytokine release (17). The increased expression of inhibitory molecules programmed death protein 1(PD-1) in CAR T cells and programmed death ligand 1 (PD-L1) in RB cells, suggests the presence of immune escape in tumor cells, and repeated exposure to antigens also inhibits the function of CAR-T cells (44). This suggests that combination therapy involving immune checkpoint inhibitors and GD2-CAR T cells may be beneficial for patients with RB.

Furthermore, Wang, K. et al. developed a local immunotherapy strategy that effectively eradicated RB cells in a mouse model without impairing vision, and even enhanced the structural and functional recovery of the retina. This was achieved by employing a hydrogel-encapsulated GD2-CAR-T releasing IL-15, which enhances the antitumor effects of GD2-CAR-Ts by sustaining their local survival (45).

### 4.2 Challenges for GD2-CAR T

Wagner, J. et al. have identified four major challenges to CAR-T therapy for solid tumors (108): Firstly, there is a limited number of targetable antigens and heterogeneous antigen expression. Heterogeneity of the structures in carbohydrate chains is a characteristic hallmark of gangliosides (127). GD2 is heterogeneously expressed in RB (7), DMG (128), pediatric high-grade gliomas (128) and NSCLC (129) tumor tissue. Secondly, there is restricted T cell viability and longevity prior to reaching tumor areas. Thirdly, T cells remain incapable of effectively navigating to tumor sites and overcoming physical barriers. Lastly, a tumor microenvironment suppresses immunity.

Among them, the lack of tumor-specific targets is a key factor hindering progress in CAR development for solid tumors. To date, clinical studies of adoptive cell therapy for NB have focused on GD2-CAR T cells. However, potential other targets or more refined CAR engineering will need to be sought out to improve the efficacy of CAR T cells for NB. Recently, Sun, M. et al. developed a CAR T therapy called CT3.28H.BBζ for children with NB, which involves a new immunotherapy target called glypican-2(GPC2) which outperformed GD-2 CAR, K666.28H.BBζ, both *in vivo* and *in vitro* trials (130). In addition, Bergaggio, E. et al. developed CAR constructs targeting the anaplastic lymphoma kinase (ALK) receptor (131), which is selectively expressed by neuroblastoma cells, and showed that ALK.CAR-Ts was as effective as GD2. CAR-Ts in a metastatic NB model. ALK.CAR-Ts were less active than GD2.CAR-Ts in cell lines with low ALK expression but high GD2 expression. Besides, ALK.CAR-Ts were more active than GD2.CAR-Ts in cell lines with low GD2 expression. Given the complementary nature of ALK.CAR-Ts and GD2.CAR-Ts, dual CAR constructs that co-target ALK and GD2 could combine the broad activity of GD2.CAR-Ts with the specificity of ALK.CAR-Ts.

### 4.3 Latest findings to improve GD2-CARs efficacy

The tonic signaling of CAR is thought to be a vital factor in controlling CAR-T efficacy. Strong CAR signaling can lead to rapid T-cell exhaustion, which can impair anti-tumor function (132). Chen, J. el al. reveal that positively charged plaques (PCPs) on the surface of CAR antigen-binding domains mediate CAR clustering and generate CAR tonic signals (133). For GD2-CAR with high tonic signals, reducing PCPs or increasing ionic strength in the culture medium during ex vivo CAR-T cell expansion minimizes spontaneous CAR activation and alleviates CAR-T cell exhaustion. This suggests that rational adjustment of PCPs to optimize tonic signaling and *in vivo* fitness of CAR-T cells is a promising design strategy for the next-generation CAR-T cells. Furthermore, they developed a bioinformatics tool (PCP score) to quantify positively charged plaques on the surface of the CAR as a predictor of CAR tonic signals. They proposed that a PCP score around 46-56 can produce optimal CAR signals. Targeting CARs at the T-cell receptor alpha constant (TRAC) gene to modify tonic signaling and phenotype could present a viable strategy to slow T-cell differentiation in the treatment of GD2+ solid tumors. Mueller, K.P. et al. developed viral-free TRAC-targeted CAR T cells for GD2+ solid tumors using CRISPR technology and found evidence of viral-free CRISPR CAR T cells induced solid tumor regression *in vivo* by reducing differentiation, tonic signaling, and exhaustion (134).

All currently commercialized CAR-T cells use viral vector-based transgene delivery (135). These therapies fail to meet clinical needs, in part due to high costs and supply chain constraints associated with manufacturing and qualifying GMP-grade vectors. Balke-Want, H. et al. demonstrated for the first time that plasmid DNA-mediated homology-independent targeted insertion (HITI) could be used for targeted insertion of a transgene into primary human T cells and demonstrated that the combination of HITI with CRISPR EnrichMENT (CEMENT) resulted in clinically relevant CAR-T cell yields, thus providing a highly efficient, genotoxic-free, clinical-scale production process (136). To transfer the “all-in-one” vector concept from T cells to NK cells for the treatment of solid malignancies, Rudek, L.S. et al. designed alpha retroviral “all-in-one” vector that combine constitutive GD2-CAR expression and inducible cytokine expression under the control of an inducible NFkB promoter element (137). This demonstrated a tightly linked induction of antigen-specific transgenes, with enhanced cytolytic potential, as evidenced by increased NK cell activation and cytokine release in NK cell subsets.

## 5 Other anti-tumor targeted immunotherapies

### 5.1 GD2 vaccines

The duration of passive immune effects triggered by anti-GD2 monoclonal antibodies is generally short-lived (138), whereas vaccination against GD2 as a form of active immunization may have a prolonged effect on tumors (139). Vaccination may specifically target tumor-associated antigens (TAAs), whereas non-specific active immunization may influence normal tissues of the body, which is an important distinction between the two (140). Since many TAAs are autoantigens, the body may develop immune tolerance, which can be effectively addressed by anti-Id Abs as an antigen-based vaccine (141). The mechanism of anti-Id antibody production is that when the antigen enters the body, it immunizes against the Ag and produces Ab1 against that Ag, which in turn can produce Ab2 against Ab1, i.e., anti-Id antibody. Additionally, portion of Ab2 can mimic the starting antigen and produce a specific immune response similar to that of the starting antigen, which is referred to as Ab2β (142). A major advantage of anti-Id vaccines is that they can be administered against antigens of non-protein origin. In a phase I clinical trial against an anti-unique antibody (TriGem), 40 out of 47 patients had hyperimmune sera that had Ab3 capable of binding specifically to GD2, and this anti-unique antibody vaccine showed a good IgG immune response as well as minimal toxicity (143). Another vaccine combines gangliosides with an immune carrier protein (KLH) in combination with an immune adjuvant thereby exerting an immune response. In a phase I trial, GD2 and GD3, i.e., GD2L and GD3L, formed from lactone, were combined with the immune carrier protein keyhole limpet hemocyanin (KLH) to form GD2L-KLH, GD3L-KLH, and finally with the immune adjuvant, OPT-821. The treatment also included oral immunostimulant, β-glucan. 12 out of 15 patients developed an antibody response to GD2 and/or GD3, and 6 of 10 evaluable patients showed disappearance of minimal residual disease (MRD) (144). In a subsequent phase II trial, the use of GD2/GD3 vaccine and oral β-glucan produced a strong antibody response in HR-NB patients with previous disease progression, and the results showed that high titers of anti-GD2-IgG1 were associated with improved survival (145).

### 5.2 Targeted nanoparticles

Nanoparticles can deliver cytotoxic drugs or RNA targets to tumor cells for therapeutic effects (**Figure 2E**). Nanoparticles for drug delivery can be made from lipids, polymers (e.g., dendrimers), organometallic compounds, and viruses (146). Most nanoparticles targeting tumors will usually consist of a polyethylene glycol (PEG) coating and a targeting ligand. Initially, the reticulo-endothelial system (RES) destroys the nanoparticles, which can be protected by the use of PEGs (147, 148). With this protection, the nanoparticles can safely reach the lesion and release the drug, increasing the accumulation in the bloodstream and at the site of the tumor. Secondly, the targeting ligands may be structures such as monoclonal antibodies (149), antibody fragments (150), aptamers (151), peptide-based targeting molecules (152) and small molecules (153), which can deliver the nanoparticles to the tumor lesion. When MicroRNA-34a is overexpressed in tumor cells, it can activate the cysteine asparaginase-mediated apoptotic pathway (149). Tivnan et al. found that anti-GD2 nanoparticle-mediated targeted delivery of miR-34a inhibited the growth of tumor cells by coupling MoAb ch14.18 to silica nanoparticles piggybacked with MicroRNA-34a (149). Adrian et al. found that neuroblastoma can down-regulate vascular endothelial growth factor A (VEGF-A) through GD2-mediated endocytosis by uptake of anti-GD2 liposome piggybacked siRNA (154). Etoposide was encapsulated into liposomes and coupled with anti-GD2mAb to form immunoliposomes, which not only targeted GD2-positive tumor cells and inhibited tumor cells proliferation, but also reduced systemic side effects (155).

## 6 Conclusion

This review briefly describes the expression of ganglioside disialoglycate (GD2) on the surface of retinoblastoma and its biological functions. We review several immunotherapeutic approaches against GD2, an anti-GD2 monoclonal antibody that has been applied in the clinical treatment of neuroblastoma and achieved good therapeutic effect, and recent research progress. Cellular immunotherapy attacks tumor cells via T cells or NK cells, which has shown promising therapeutic effects in the treatment of B-cell malignancies. However, the current research on GD2 is not yet available good therapeutic effect, while the treatment for solid tumors is currently in the stage of preclinical research and clinical trials. The GD2 vaccine, an active immunotherapy, has demonstrated favorable antibody responses in clinical trials. Targeted nanoparticles can deliver cytotoxic drugs or RNA to tumor cells via antibodies and other substances.
